# Dynamical analysis of long-wave phenomena for the nonlinear conformable space-time fractional (2+1)-dimensional AKNS equation in water wave mechanics

**DOI:** 10.1016/j.heliyon.2020.e05276

**Published:** 2020-10-23

**Authors:** Nur Hasan Mahmud Shahen, Foyjonnesa, Md. Habibul Bashar, Md. Shuzon Ali, Abdulla - Al - Mamun

**Affiliations:** a European University of Bangladesh, Dhaka 1216, Bangladesh; b University of Rajshahi, Rajshai 6205, Bangladesh; c Bangabandhu Sheikh Mujibur Rahman Science and Technology University, Gopalgonj 8100, Bangladesh; d Islamic University, Kushtia 7003, Bangladesh

**Keywords:** Applied mathematics, Nonlinear physics, Space-time fractional (2+1)-dimensional AKNS equation, Conformable derivative, The advanced exp(-ϕ(ξ))-expansion method

## Abstract

The main intension of this paper is to extract new and further general analytical wave solutions to the (2 + 1)-dimensional fractional Ablowitz-Kaup-Newell-Segur (AKNS) equation in the sense of conformable derivative by implementing the advanced exp(-ϕ(ξ))-expansion method. This method is a particular invention of the generalized exp(-ϕ(ξ))-expansion method. By the virtue of the advanced exp(-ϕ(ξ))-expansion method, a series of kink, singular kink, soliton, combined soliton, and periodic wave solutions are constructed to our preferred space time-fractional (2 + 1)- dimensional AKNS equation. An extensive class of new exact traveling wave solutions are transpired in terms of, hyperbolic, trigonometric, and rational functions. To express the underlying propagated features, some attained solutions are exhibited by making their three-dimensional (3D), two-dimensional (2D) combined, and 2D line plot with the help of computational packages MATLAB. All plots are given to show the proper wave features through the founded solutions to the studied equation with particular preferring of the selected parameters. Moreover, it may conclude that the attained solutions and their physical features might be helpful to comprehend the water wave propagation in water wave mechanics.

## 1 Introduction

As of late, nonlinear fractional partial differential equations (FPDEs) are one of the progressing fields of applied mathematics, computational mathematics, and mathematical physics whose thought was first introduced in 1695 [1]. It is widely used to comprehend complex physical phenomena of applied science, fractional dynamics, plasma physics, chemical physics, astrophysics, mechanical engineering, neural material science, strong state material science, stochastic dynamical system, nonlinear optics, geo-optical filaments, and so on [2, 3, 4].

In the past few decades, a lot of concern has been executed to find the new and further exact solutions of space-time fractional nonlinear partial differential equations (PDEs) by introducing several types of research. With the assists of potential computer programming software, they have been appointed for researching some appropriate solutions to the nonlinear space-time FPDEs by executing powerful techniques, namely the Legendre collocation method [5], the Adomian decomposition way [6, 7], the finite difference method [8], the ${\text{G}}^{\prime }{\text{/G}}^{2}$-expansion scheme [9], the Hirota's bilinear method [10, 11, 12, 13], the ansatz manner [14, 15], the simplified form of bilinear method [16], the improved tanh-system [17], the residual power series method [18], the exp-function method [19, 20], the exp(-ϕ(ξ))-expansion method [21], the generalized Kudryashov method [22], the advanced exp(-ϕ(ξ))-expansion method [23, 24], the (${\text{G}}^{\prime }\text{/G}$, 1/G)-expansion strait [25, 26, 27, 28, 29, 30], the sine-Gordon expansion scheme [31, 32], the ${\text{G}}^{\prime }\text{/G}$-expansion method [33], the advanced exponential expansion method [24], the generalized tanh-coth method [34], the generalized exp(-ϕ(ξ))-expansion method [35], the extended trial equation method [36], and so on.

This study mainly focusses on the dynamical analysis of (2 + 1)-dimensional space-time fractional AKNS equation [33, 37] with the application of the advanced exp(-ϕ(ξ))-expansion method [23, 24]. Recently, Bashar and Roshid [23], and Rahhman et al. [24] have exposed this method to some fractional and non-fractional PDEs. Rahhman et al. [24] didn't give any fruitful discussion about FPDEs in the sense of conformable derivative with our preferred method. Whereupon the obtained exact solutions of their studies [23, 24] are not novel in the sense of conformable time fractional derivative.

Considering this fact, we firmly intended ourselves to find out the exact solution of the nonlinear conformable space-time fractional (2 + 1)-dimensional AKNS water wave condition with the aid of the advanced exp(-ϕ(ξ))-expansion scheme. Here it's important to know that the water wave equations such as (2 + 1)-dimensional coupled Davey-Stewartson equation (DSE), (2 + 1)- dimensional AKNS equation, Regularized long wave equation (RLWE), etc. are now the topic of analytical phenomena in mathematical physics [37]. Many real type nonlinear features derived in water wave equations and its deal with the ocean waves from storms, with waves of flood in rivers, with the wave of ship on water, with the free oscillations of closed waters such as harbors and lakes [38].

Let us consider the following space-time fractional (2 + 1)-dimensional AKNS water wave equation [33]:

(1) $$4\;{\text{M}}_{x}^{\beta }\;{\text{M}}_{t}^{\alpha }\;u+{\text{M}}_{x}^{\beta }{\text{M}}_{x}^{\beta }{\text{M}}_{x}^{\beta }\;{\text{M}}_{\text{y}}^{\theta }\;u+8\;{\text{M}}_{x}^{\beta }\;{\text{M}}_{\text{y}}^{\theta }\;u\;{\text{M}}_{x}^{\beta }u+4\;{\text{M}}_{\text{y}}^{\theta }\;u\;{\text{M}}_{x}^{\beta }{\text{M}}_{x}^{\beta }\;u-a\;{\text{M}}_{x}^{\beta }{\text{M}}_{x}^{\beta }\;u=0,$$

where a≠0, is a free parameter.

Our preferred (2 + 1)-dimensional AKNS water wave condition is one of the effective prominent physical models in water wave mechanics [37, 38, 39, 40, 41]. From the implicit parameter-dependent symmetry limitations of the KP equation in 1997, Lou and Hu [40] have explored this (2 + 1)-dimensional AKNS water wave equation. Previous several studies have been investigating the exact solutions of the AKNS equation by the virtue of some powerful techniques, such as the Hirota's bilinear method, the anstatz method [10, 14, 15], the Lie-symmetry method [42], etc. But with the sense of comformable derivative, there is no pragmatic studies are not found yet about our advanced exp(-ϕ(ξ))-expansion method to investigate the space-time fractional (2 + 1)-dimensional AKNS water wave equation. Moreover, our mentioned method is flourishing and gives a functional accurate form of exact solutions to the space-time FPDEs. Very recently, Hafez et al. [35] introduced a so-called method, namely generalized exp(-ϕ(ξ))-expansion method in the sense of Jumarie's modified Riemann-Liouville derivatives by taking the auxiliary nonlinear ODE of the form $\phi^{\prime }\;\left(\xi \right)-\lambda \;\exp\;\left(\phi \;\left(\xi \right)\right)-\mu \;\exp\;\left(-\;\phi \;\left(\xi \right)\right)=r$. It is noteworthy that, the important idea of our mentioned advanced exp(-ϕ(ξ))-expansion method is too explicit the exact solutions of FPDEs that satisfying the auxiliary nonlinear ODE of the form $\phi^{\prime }\;\left(\xi \right)-\lambda \;\exp\;\left(\phi \;\left(\xi \right)\right)-\mu \;\exp\;\left(-\;\phi \;\left(\xi \right)\right)=0,$ by taking r=0 in the generalized exp(-ϕ(ξ))-expansion method [35] in the sense of conformable derivative. Where λ and μ are real parameters and comparatively our mentioned auxiliary form provides much better comprehensive solutions to the FPDEs as well as has a structural physical explanation than the study of Hafez et al. [35].

Bashar and Roshid [23], and Rahhman et al. [24] took this ODE auxiliary form as $\phi^{\prime }\;\left(\xi \right)+\lambda \;\exp\;\left(\phi \;\left(\xi \right)\right)+\mu \;\exp\;\left(-\;\phi \;\left(\xi \right)\right)=r$. The main favor of our mentioned method over the other existing methods [24, 35] is that it gives some direct and succinct form of exact traveling wave solution as well as, very efficient and friendly applicable in introducing of explicit traveling wave solutions to FPDEs, arises in engineering and mathematical physics. With the assists of computational software MATLAB, we have represented the obtained solutions by taking fruitful values of the included parameters by delineating sketches to understand the physical explanation properly.

The study is decorated in the subsequent: In section 2, the narration of the conformable space-time fractional differential equation is presented. In section 3, the advanced exponential extension scheme has been talked about. In segment 4, we apply this mentioned scheme to the conformable space-time fractional (2 + 1)-dimensional AKNS equation. In section 5, results and discussion, in section 6, conclusions are conferred.

## 2 Preliminaries and procedures

### 2.1 Definition and some aspects of conformable fractional derivative

Khalil et al. [43] firstly explored the conformable fractional derivative with the operator of a limit.

**Definition:** If f:(0,∝)→ℜ, then the conformable derivative in fractional sense of f order δ is defined as

$M_{t}^{\delta }f\left(t\right)=\underset{\varepsilon \to 0}{\lim}\left(\frac{f\;\left(t+\varepsilon \;t^{1-\delta }\right)-f\left(t\right)}{\varepsilon }\right)$ for all t>0,0<δ≤1.

Later, Abdeljawad [44] has also proposed chain rule, Gronwalls inequality, exponential functions, integration by parts, Taylor power series expansions and Laplace transform for conformable derivative in fractional way. The definition of conformable fractional derivative can easily defeated the complexity of exiting modified Riemann-Liouville derivative definition [45].

**Theorem 1:** Let δ∈(0,1], and f=f(t),g=g(t) be δ-conformable differentiable at a point t>0, then:

(i) $M_{t}^{\delta }\left(cf+dg\right)=cM_{t}^{\delta }f+dM_{t}^{\delta }g,\;\text{for}\;\text{all}\;c,\;d\in ℜ$,
(ii) $M_{t}^{\delta }\left(t\gamma \right)=\gamma \;t^{\gamma -\delta },\;\text{for}\;\text{all}\;\gamma \in ℜ$,
(iii) $M_{t}^{\delta }\left(fg\right)=gM_{t}^{\delta }\left(f\right)+f\;M_{t}^{\delta }\left(g\right)\;,\;$
(iv) $M_{t}^{\delta }\left(\frac{f}{g}\right)=\frac{gM_{t}^{\delta }\left(f\right)-f\;M_{t}^{\delta }\left(g\right)}{g^{2}}.$

Moreover, if the function f is differentiable, then $M_{t}^{\delta }(f\left(t\right))=\;t^{1-\delta }\frac{df}{dt}.$

**Theorem 2:** Let f:(0,∝)→ℜ, be a function such that f is differentiable and δ-conformable differentiable. Also, let g be a differentiable function discussed in the range of function f. Then

$$M_{t}^{\delta }\left(fog\right)\;\left(t\right)=t^{1-\delta }g{\left(t\right)}^{\delta -1}\;g^{\prime }\left(t\right)\;M_{t}^{\delta }{(f\left(t\right))}_{t=g\left(t\right)}\;,$$

where prime denotes the classical derivatives with respect to t.

## 3 The advanced exp(−ϕ(ξ))-expansion method

In this section, we discuss our mentioned exp(-ϕ(ξ))-expansion method step by step in details. Consider a nonlinear partial differential equation in the following form,

(2) $$ℜ\;\left(U,\;U_{xx}\;,\;U_{xy}\;,U_{xz}\;\;,U_{xxx}\;,\;U_{xtt\;},\ldots \;\ldots \right)=0\;,$$

where U=U(x,y,z,t) is an unknown function, ℜ is a polynomial of U, it's a different type of partial derivatives, in which the nonlinear terms and the highest order derivatives are included.

**Step-1.** Now we consider a transformation variable to convert all independent variable into one variable, such as

(3) U(x,y,z,t)=u(ξ),ξ=kx+ly+mz±Vt.

By utilizing this variable Eq. (3) permits us reducing Eq. (2) in an ODE for u(x,y,z,t)=u(ξ) into the form

(4) $$P\left(u,\;u^{\prime },\;u^{\prime \prime },\cdots \;\cdots \;\cdots \right)=0$$

**Step-2.** Let us assume that the solution of ODE Eq. (4) can be expressed by a polynomial in exp(−ϕ(ξ)) as the form

(5) $$u=\overset{N}{\underset{i=0}{\sum }}\;A_{i}\;exp(-\phi (\xi ))i,\;\;\;A_{N}\neq 0,$$

where the positive integer N can be obtained by balancing the highest order derivatives to the highest order nonlinear terms appear in Eq. (4).

And the derivative of ϕ(ξ) satisfies the ODE in the following form

(6) $$\phi^{\prime }\left(\xi \right)-\lambda \;\exp\;\left(\phi \;\left(\xi \right)\right)-\mu \;\exp\;\left(-\phi \;\left(\xi \right)\right)=0,$$

then the solutions of ODE Eq. (6) are

**Case I:**

Hyperbolic function solution (when λμ<0):

$$\phi \left(\xi \right)=\ln(\sqrt{\frac{\lambda }{-\mu }}\tanh(\sqrt{-\lambda \mu }\left(\xi +C\right)))$$

$$\text{and}\;\phi \left(\xi \right)=\ln(\sqrt{\frac{\lambda }{-\mu }}\coth(\sqrt{-\lambda \mu }\left(\xi +C\right)))$$

**Case II:**

Trigonometric function solution (when λμ>0):

$$\phi \left(\xi \right)=\ln(\sqrt{\frac{\lambda }{\mu }}\tan(\sqrt{\lambda \mu }\left(\xi +C\right)))$$

$$\text{and}\;\phi \left(\xi \right)=\ln(-\sqrt{\frac{\lambda }{\mu }}\cot(\sqrt{\lambda \mu }\left(\xi +C\right)))$$

**Case III:**

when μ>0 and λ=0

$$\phi (\xi )=\ln\left(\frac{1}{-\mu (\xi +C)}\right)$$

**Case IV:**when μ=0 and λ∈ℜ

ϕ(ξ)=ln(λ(ξ+C)).

Where C is an integrating constant and λμ<0orλμ>0 depends on sign of μ.

**Step-3.** By substituting Eq. (5) into Eq. (4) and utilizing the Eq. (6), collecting all like type order of exp(−mϕ(ξ)),m=0,±1,±2,±3,.... together, then we execute a polynomial form exp(−mϕ(ξ)) and equating each coefficients of this polynomial equal to zero, yields a set of algebraic system.

**Step-4.** Assume the estimation of the constants can be gotten by fathoming the mathematical conditions got in step 3. Substituting the estimations of the constants together with the arrangements of Eq. (6), we will acquire new and far reaching precise traveling wave arrangements of the nonlinear development Eq. (2).

## 4 Application of (2 + 1)-dimensional AKNS equation

Considering the conformable space-time fractional AKNS equation as follows (see for example [33]):

(7) $$\begin{array}{l} 4\;{\text{M}}_{x}^{\beta }\;{\text{M}}_{t}^{\alpha }\;u+{\text{M}}_{x}^{\beta }{\text{M}}_{x}^{\beta }{\text{M}}_{x}^{\beta }\;{\text{M}}_{\text{y}}^{\theta }\;u+8\;{\text{M}}_{x}^{\beta }\;{\text{M}}_{\text{y}}^{\theta }\;u\;{\text{M}}_{x}^{\beta }u+4\;{\text{M}}_{\text{y}}^{\theta }\;u\;{\text{M}}_{x}^{\beta }{\text{M}}_{x}^{\beta }\;u-a\;{\text{M}}_{x}^{\beta }{\text{M}}_{x}^{\beta }\;u=0,\\ 0<\alpha \leq 1,\;0<\beta \leq 1,\;0<\theta \leq 1, \end{array}\}$$

(8) $$\text{where}\;u\;\left(x,\;y,\;t\right)=u\;\left(\xi \right),\;\xi =w\frac{t^{\alpha }}{\alpha }+r\frac{x^{\beta }}{\beta }+s\frac{y^{\theta }}{\theta },\;\text{and}\;\text{a}\;\text{is}\;\text{a}\;\text{real}\;\text{parameter.}$$

By utilizing the wave transformation of Eq. (8), we get the transformation form of Eq. (7) into the following form of ordinary differential equation.

(9) $$4wru^{\prime \prime }+r^{3}su^{4}+8r^{2}su^{\prime \prime }u^{\prime }+4r^{2}su^{\prime }u^{\prime \prime }-ar^{2}u^{\prime \prime }=0.$$

Now integrating Eq. (3) with respect to ξ with zero constant of integration, we get

(10) $$\left(4w-ar\right)\;u^{\prime }+r^{2}su^{\prime \prime \prime }+6rs\;{\left(u^{\prime }\right)}^{2}=0,$$

where primes refer to the differentiation with respect to ξ. By balancing nonlinear term and highest order derivative term in Eq. (10), we can obtain the value of N as 1, so the Eq. (5) takes the following form:

(11) $$u\;\left(\xi \right)=A_{0}+A_{1}\;\exp\;\left(-\phi \left(\xi \right)\right)\;.$$

Differentiating Eq. (11) with respect to ξ and substituting the value of $u,\;u^{\prime },\;u^{\prime \prime },\;u^{\prime \prime \prime }$ into the Eq. (10) and finally equating the coefficients of $\;{exp(-\varphi (\xi ))}^{i}$ equal to zero, where i=0,±1,±2,⋯⋯; we get some of following system of equations:

$$-2r^{2}sA_{1}\lambda \mu^{2}+6rs{A_{1}}^{2}\mu^{2}+A_{1}\mu \;a\;r-4A_{1}\mu \;w=0,$$

$$-8\lambda^{2}\mu \;r^{2}sA_{1}+12\lambda \mu \;r\;s+{A_{1}}^{2}+a\lambda rA_{1}-4\lambda wA_{1}=0,$$

$$-6\lambda^{3}r^{2}sA_{1}+6\lambda^{2}rs{A_{1}}^{2}=0.$$

Solving this system of equations, we get only one set solutions

**Set 1:**

$$w=\lambda \mu \;r^{2}s+\frac{1}{4}ar,\;A_{0}=A_{0},\;A_{1}=\lambda r.$$

**Case 1:** When λμ<0, we get following hyperbolic solutions:

**Family 1**

$$\begin{array}{l} \begin{array}{cc} u_{1}\;\left(x,\;y,\;t\right)=A_{0} & +\frac{\lambda r\;\cosh\left(\sqrt{-\lambda \mu }\;\xi \right)\cosh\left(\sqrt{-\lambda \mu }\;C\right)}{\sqrt{-\frac{\lambda }{\mu }}\;\left(\sinh\left(\sqrt{-\lambda \mu }\;\xi \right)\cosh\left(\sqrt{-\lambda \mu }\;C\right)+\cosh\left(\sqrt{-\lambda \mu }\;\xi \right)\sinh\left(\sqrt{-\lambda \mu }\;C\right)\right)}\\ & +\frac{\lambda r\;\sinh\left(\sqrt{-\lambda \mu }\;\xi \right)\sinh\left(\sqrt{-\lambda \mu }\;C\right)}{\sqrt{-\frac{\lambda }{\mu }}\;\left(\sinh\left(\sqrt{-\lambda \mu }\;\xi \right)\cosh\left(\sqrt{-\lambda \mu }\;C\right)+\cosh\left(\sqrt{-\lambda \mu }\;\xi \right)\sinh\left(\sqrt{-\lambda \mu }\;C\right)\right)}. \end{array}\\ \begin{array}{cc} u_{2}\;\left(x,\;y,\;t\right)=A_{0} & +\frac{\lambda r\;\sinh\left(\sqrt{-\lambda \mu }\;\xi \right)\cosh\left(\sqrt{-\lambda \mu }\;C\right)}{\sqrt{-\frac{\lambda }{\mu }}\;\left(\cosh\left(\sqrt{-\lambda \mu }\;\xi \right)\cosh\left(\sqrt{-\lambda \mu }\;C\right)+\sinh\left(\sqrt{-\lambda \mu }\;\xi \right)\sinh\left(\sqrt{-\lambda \mu }\;C\right)\right)}\\ & +\frac{\lambda r\;\cosh\left(\sqrt{-\lambda \mu }\;\xi \right)\sinh\left(\sqrt{-\lambda \mu }\;C\right)}{\sqrt{-\frac{\lambda }{\mu }}\;\left(\cosh\left(\sqrt{-\lambda \mu }\;\xi \right)\cosh\left(\sqrt{-\lambda \mu }\;C\right)+\sinh\left(\sqrt{-\lambda \mu }\;\xi \right)\sinh\left(\sqrt{-\lambda \mu }\;C\right)\right)}. \end{array} \end{array}$$

Where $\xi =\frac{rx^{\beta }}{\beta }+\frac{sy^{\theta }}{\theta }+\frac{\left(\lambda \mu \;r^{2}s+\frac{1}{4}\mathrm{ar}\right)t^{\alpha }}{\alpha }$, Cis an arbitary constant.

**Case 2:** When λμ>0, we get following trigonometric solutions:

**Family 2**

$$u_{3}\;\left(x,\;y,\;t\right)=\frac{-\lambda r\;\tan\left(\sqrt{\lambda \mu }\;C\right)\tan\left(\sqrt{\lambda \mu }\;\xi \right)+A_{0}\sqrt{\frac{\lambda }{\mu }}\;\tan\left(\sqrt{\lambda \mu }\;C\right)+A_{0}\sqrt{\frac{\lambda }{\mu }}\;\tan\left(\sqrt{\lambda \mu }\;\xi \right)+\lambda r}{\sqrt{\frac{\lambda }{\mu }}\;\left(\tan\left(\sqrt{\lambda \mu }\;C\right)+\tan\left(\sqrt{\lambda \mu }\;\xi \right)\right)}.\;u_{4}\;\left(x,\;y,\;t\right)=A_{0}-\frac{\lambda rcot\left(\sqrt{\lambda \mu }\;C\right)}{\sqrt{\frac{\lambda }{\mu }}\;\left(-1+cot\left(\sqrt{\lambda \mu }\;C\right)cot\left(\sqrt{\lambda \mu }\;\xi \right)\right)}-\frac{\lambda rcot\left(\sqrt{\lambda \mu }\;\xi \right)}{\sqrt{\frac{\lambda }{\mu }}\;\left(-1+cot\left(\sqrt{\lambda \mu }\;C\right)cot\left(\sqrt{\lambda \mu }\;\xi \right)\right)}.\;$$

Where $\xi =\frac{rx^{\beta }}{\beta }+\frac{sy^{\theta }}{\theta }+\frac{\left(\lambda \mu \;r^{2}s+\frac{1}{4}ar\right)\;t^{\alpha }}{\alpha }$, Cis an arbitary constant.

**Case 3:** When μ=0,λ∈ℜ we get following solutions:

**Family 3**

$$u_{5}\;\left(x,\;y,\;t\right)=\frac{A_{0}C}{\xi +C}+\frac{A_{0}\xi }{\xi +C}+\frac{r}{\xi +C},$$

where $\xi =\frac{rx^{\beta }}{\beta }+\frac{sy^{\theta }}{\theta }+\frac{\left(\lambda \mu \;r^{2}s+\frac{1}{4}ar\right)\;t^{\alpha }}{\alpha }$, Cis an arbitary constant.

**Case 4:**

When λ=0, and μ>0, the solution cannot be determined, so we can neglect this case.

## 5 Results and discussion

**Figure 1:**
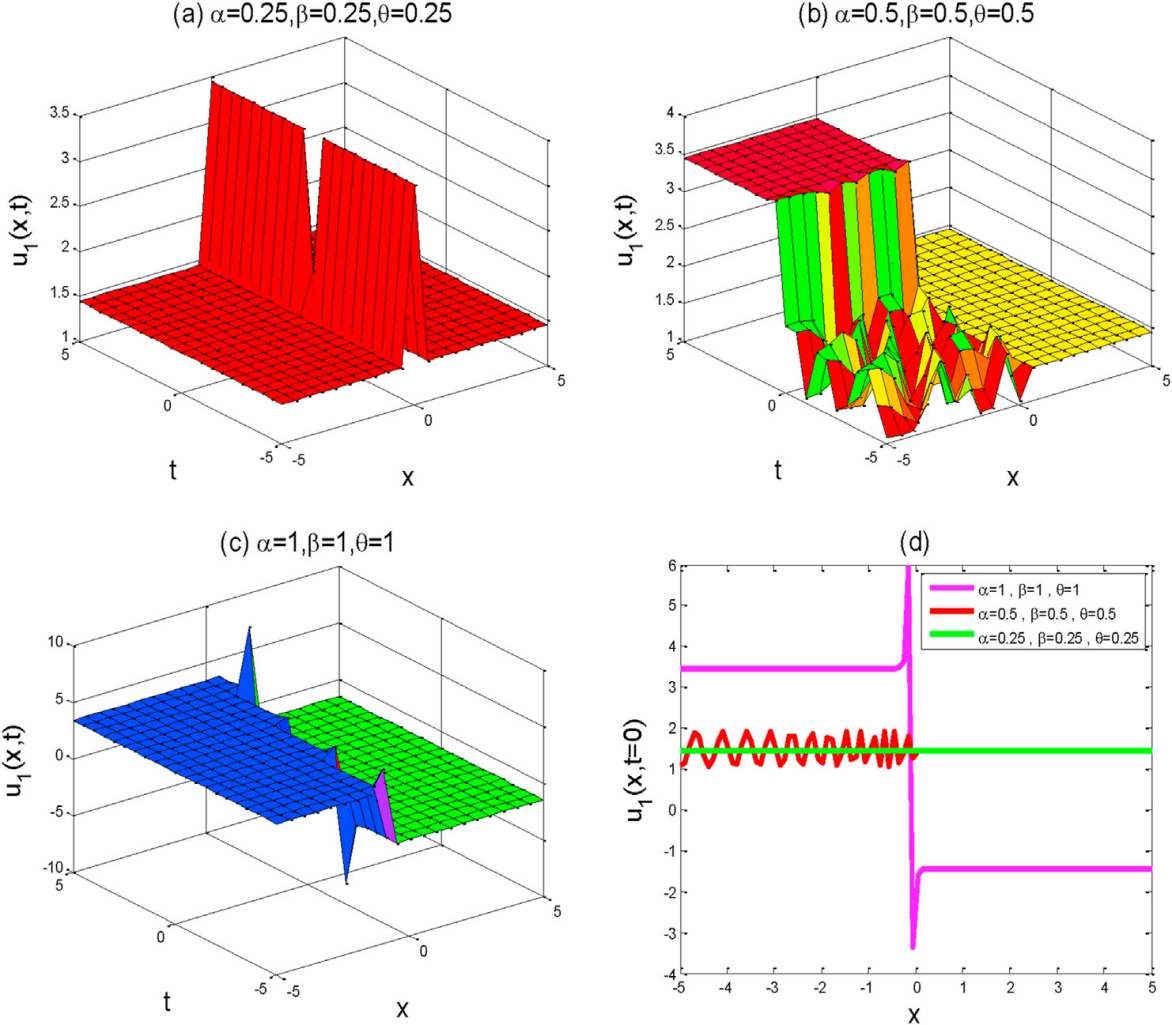
The above figures represent the solution shape of $u_{1}\;\left(x,\;y,\;t\right)$. For each graph we choose the space-time fractional values of  (a) α=0.25 , β=0.25,θ=0.25,  (b) α=0.5 ,β=0.5, θ=0.5,  and (c)α=1 , β=1, θ=1 . First three a,b,c figures show the 3D plot for y=0 and the fourth figure d shows the 2D combined line plot for t=0.

**Figure 2:**
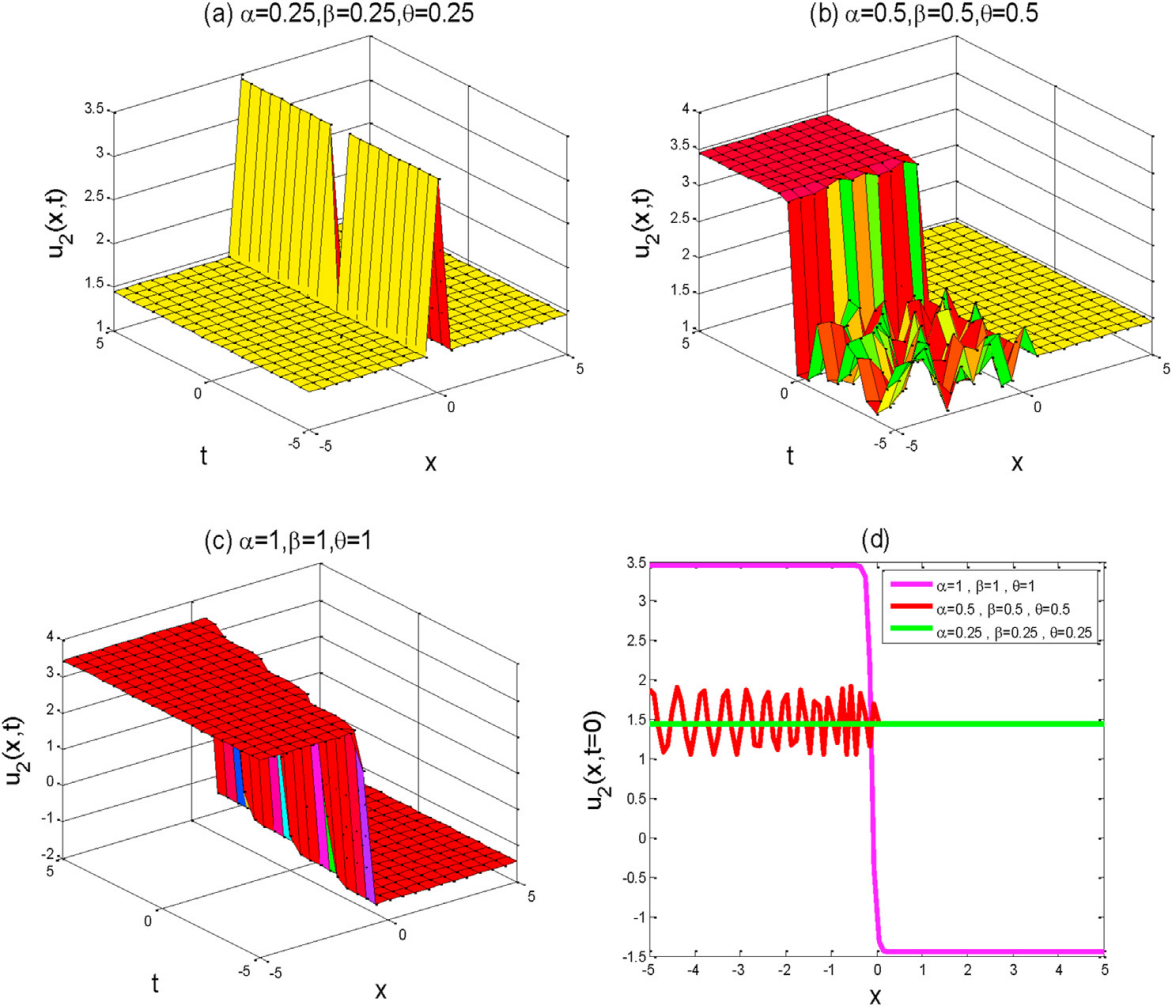
Above figures represent the solution graph of $u_{2}\;\left(x,\;y,\;t\right)$. The first three a,b,c figures show the 3D plot for y=0 and the fourth figure d shows the 2D combined line plot for t=0.

**Figure 3:**
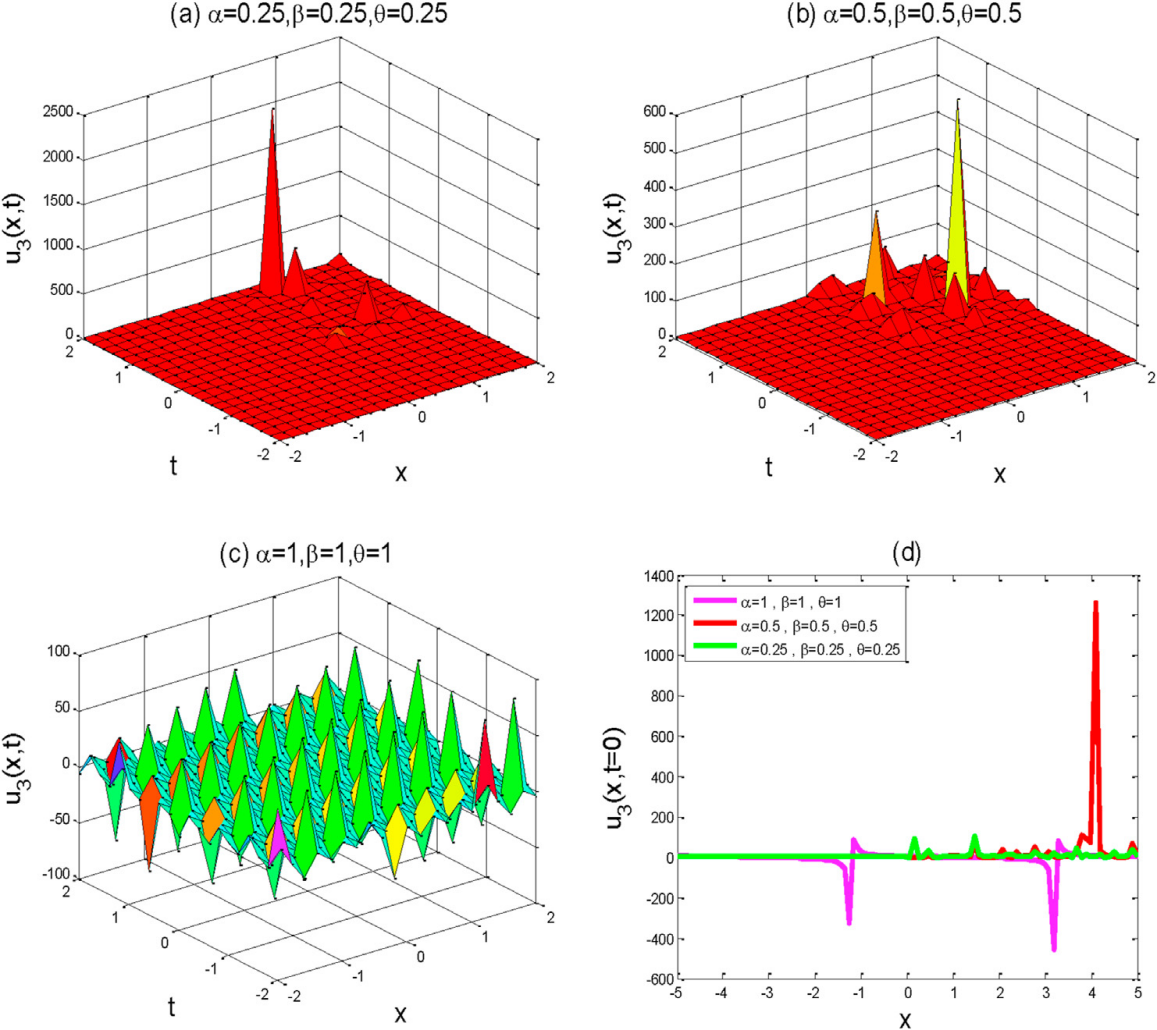
Above figures represent the solution graph of $u_{3}\;\left(x,\;y,\;t\right)$. The first three a,b,c figures show the 3D plot for y=0 and the fourth figure d shows the 2D combined line plot for t=0.

**Figure 4:**
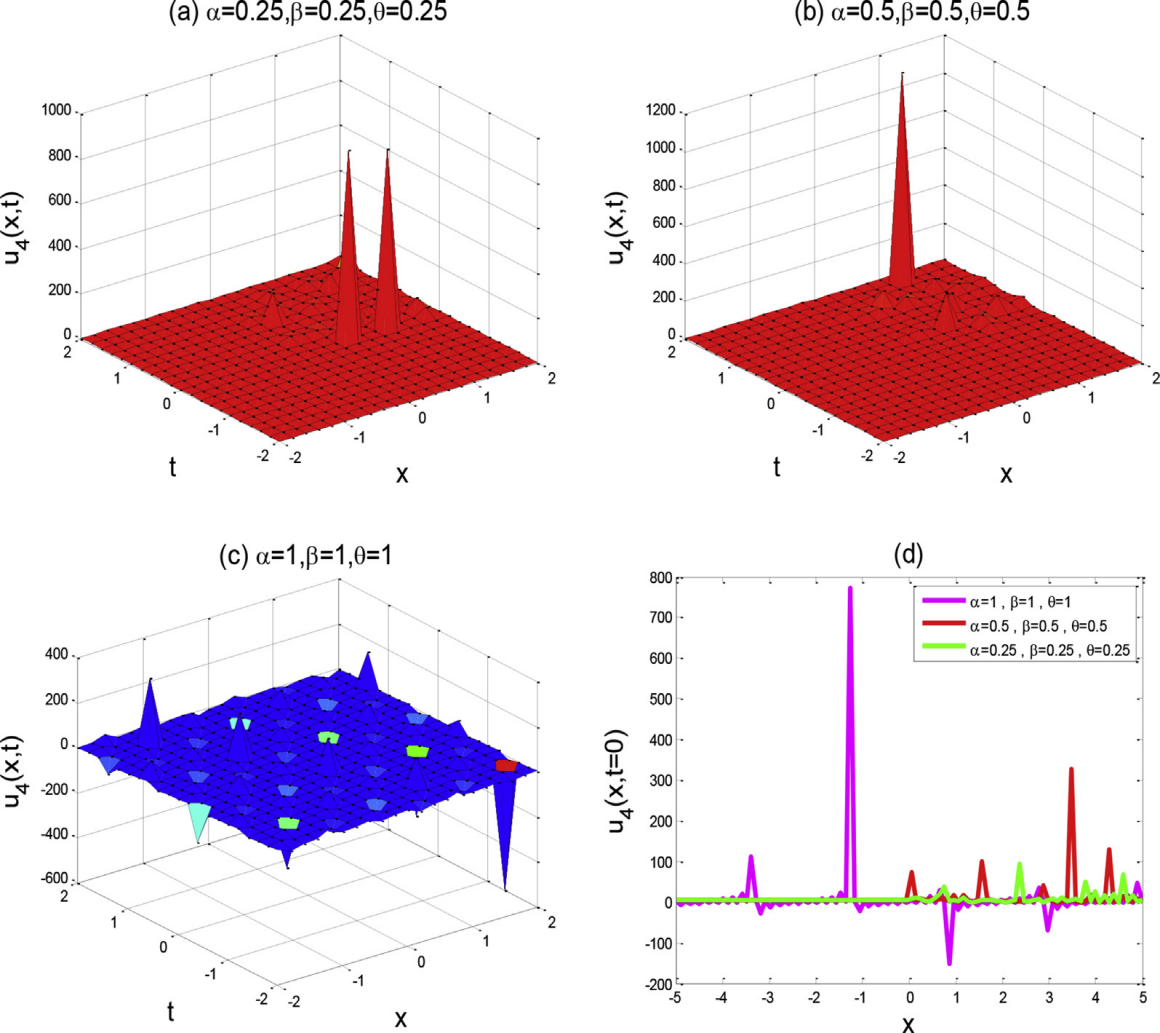
Above figures represent the solution graph of $u_{4}\;\left(x,\;y,\;t\right)$. The first three a,b,c figures show the 3D plot for y=0 and the fourth figure d shows the 2D combined line plot for t=0.

**Figure 5:**
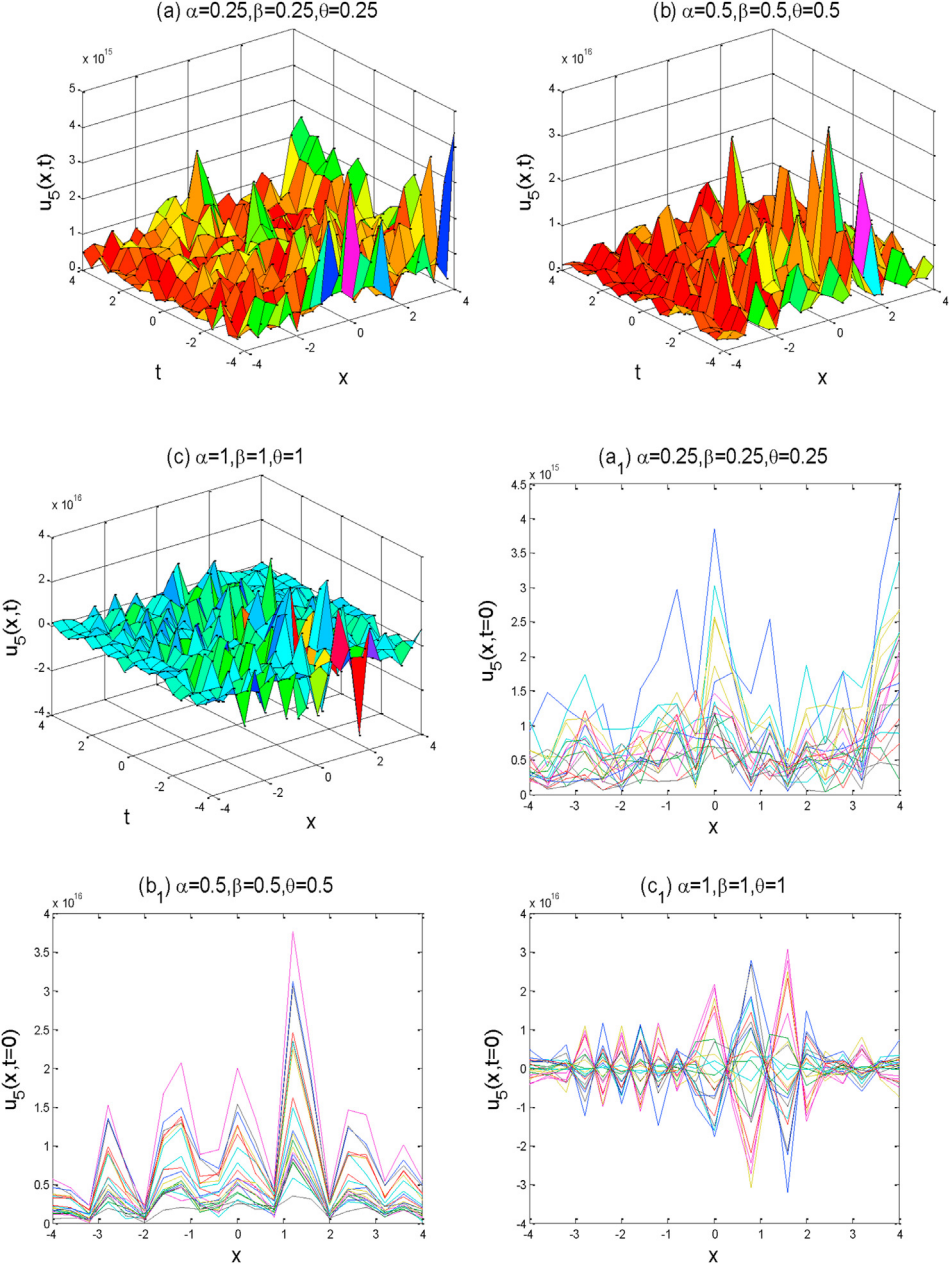
Above figures represent the solution graph of $u_{5}\;\left(x,\;y,\;t\right)$. In every set of above Figures 1, 2, 3, and 4 the 2D combined line plots are used to show the high frequencies and very small amplitude of the exact wave responses very clearly. In this figure, we added some additional individual 2D line plot of a,b, and c which is denoted by $a_{1},\;b_{1},\;\text{and}\;c_{1}$ respectively.

In this section, we will discuss the physical interpretation and graphical representation of the obtained exact solutions of the (2 + 1)-dimensional AKNS equation. All exact solutions have been derived from the mentioned equation by the aid of computational software Maple-17. The graphical illustration is the absolute way to know the real physical signs of any real-life problems. With the help of computational software MATLAB, we have shown the graphical representation of some obtained solutions in the sense of conformable fractional derivative by using some potential fractional values of α,β,andθ. We utilized suitable values to the unknown parameters in order to visualize the real futures of the derived solutions. The obtained solutions have been sketched the studied equations which are shown in Figures 1, 2, 3, 4, and 5.

### 5.1 Physical explanation

Around there, we will discuss the physical explanation of the exact solutions of (2 + 1)-dimensional AKNS equation by using the advanced exp(-ϕ(ξ))-expansion method. The obtained solution $u_{1}\;\left(x,\;y,\;t\right)$ and $u_{2}\;\left(x,\;y,\;t\right)$ are hyperbolic function solutions, $u_{3}\;\left(x,\;y,\;t\right)$ and $u_{4}\;\left(x,\;y,\;t\right)$ are trigonometric function solutions, and $u_{5}\;\left(x,\;y,\;t\right)$ is rational function solution. Fig. (1) represents the kink solution with the parameters $\lambda =3\text{, }\mu =-2\text{, C}=0.5\text{, s}=1\text{,r}=1\text{,}{\text{A}}_{\text{0}}=1,$y=0,a=1 and the fractional values of α=0.25,0.5,1,β=0.25,0.5,1 and θ=0.25,0.5,1 respectively within the interval −5≤x≤5 and −5≤t≤5. We observed that when the fractional order of derivatives α,β,andθ are increased, the kink shape is closer to the known kink shape as the velocity of the propagation wave decreases. The kink shape changes its height with the change of fractional order of derivative α,β,andθ, respectively. Here the change of free parameter a with 1to−1 or otherwise Figure 1 remains unchanged i.e. the exact solution $u_{1}\;\left(x,\;y,\;t\right)$ has no dynamical variation within the changes. Figure 2 represents the singular kink solution type of exact traveling wave solution with the parameters $\lambda =3\text{, }\mu =-2\text{, C}=0.5\text{, s}=1\text{,r}={1\text{,A}}_{\text{0}}=1,$y=0,a=1 and the same fractional parameters α,β,andθ within the interval −5≤x≤5 and −5≤t≤5. Here the change of free parameter a with 1to−1 or otherwise Figure 2 remains unchanged i.e. the function solution $u_{2}\;\left(x,\;y,\;t\right)$ has no dynamical change also. Figure 3 represents the graph of periodic shape of $u_{3}\;\left(x,\;y,\;t\right)$ for the parameters $\lambda =3\text{, }\mu =2\text{, C}=0.5\text{, s}=1\text{,r}=1\text{,}{\text{A}}_{\text{0}}=1,$y=0,a=1 and α=1 , β=1, θ=1  within the interval −2≤x≤2 and −2≤t≤2. Here with the decrease of fractional order of derivatives α,β,andθ, the periodic shape changes its height as well as the periodic shape forward to the singular soliton shape. Figure 4 represents the periodic function solution of $u_{4}\;\left(x,\;y,\;t\right)$ for the parameters λ=3, μ=2, C=0.5, s=1,r=1,${\text{A}}_{\text{0}}=1,y=0,\;a=1$ and α=1 , β=1, θ=1  within the interval −2≤x≤2 and −2≤t≤2. Here the changes of free parameter a with 1to−1 or otherwise Figure 4 remains unchanged but at the change of fractional parameters the periodic shape changes its height and turn into the singular soliton shape. Figure 5 represents the singular kink solution shape of $u_{5}\;\left(x,\;y,\;t\right)$ for the parameters $\lambda =\text{0, }\mu =2\text{, C}=0.5\text{, s}=1\text{,r}=1\text{,}{\text{A}}_{\text{0}}=1,$y=0,a=1 within the interval −4≤x≤4 and −4≤t≤4. Here with the changes of free parameter a with 1to−5 the singular kink shape turn into a combined soliton shape.

### 5.2 Graphical representation

In this section, we will show the graphical patterns of our gained solutions of the space-time fractional (2 + 1)-dimensional AKNS equation. The solutions are fully derived with the aid of computational software MAPLE in terms of the hyperbolic, trigonometric, and rational function. To plot graphs we have used computational software MATLAB. All exact solutions are shown in MATLAB 3D, 2D line and combined line plot for the comprehensive physical explanation.

## 6 Conclusion

In this study, we have established some new solutions includes kink, soliton, combined soliton, singular-kink, and periodic wave to the (2 + 1)-dimensional AKNS water wave equation and showed the dynamical behavior of the obtained solutions with the changes of free parameter and fractional derivative orders. It is important to note that the novel solutions of the space-time fractional (2 + 1)-dimensional AKNS equation have not been exposed by advanced exp(-ϕ(ξ))-expansion in previous literature. Thus, it can be claimed that the obtained solutions are novel in the sense of conformable derivative to the space time fractional AKNS equation. Finally, it can be concluded that our preferred method is effective, reliable, authentic, conformable, powerful and gives ample consistent solutions to space-time fractional nonlinear PDEs arise in the field of mathematics, applied mathematics, nonlinear dynamics, mathematical physics, water wave mechanics, engineering, and so on.

## Declarations

### Author contribution statement

Nur Hasan Mahmud Shahen: Conceived and designed the experiments; Performed the experiments; Analyzed and interpreted the data; Contributed reagents, materials, analysis tools or data; Wrote the paper.

Foyjonnesa: Conceived and designed the experiments; Performed the experiments; Analyzed and interpreted the data; Wrote the paper.

Md. Habibul Bashar, Md. Shuzon Ali: Analyzed and interpreted the data.

Abdulla - Al - Mamun: Performed the experiments.

### Funding statement

This research did not receive any specific grant from funding agencies in the public, commercial, or not-for-profit sectors.

### Competing interest statement

The authors declare no conflict of interest.

### Additional information

No additional information is available for this paper.
